# Littoral Cell Angioma in a Patient with Crohn's Disease

**DOI:** 10.1155/2015/474969

**Published:** 2015-01-29

**Authors:** Joel Johansson, Bergthor Björnsson, Simone Ignatova, Per Sandström, Mattias Ekstedt

**Affiliations:** ^1^Department of Gastroenterology and Department of Clinical and Experimental Medicine, Linköping University, 58185 Linköping, Sweden; ^2^Department of Surgery, County Council of Östergötland, 58185 Linköping, Sweden; ^3^Division of Surgery, Department of Clinical and Experimental Medicine, Faculty of Health Sciences, Linköping University, 581 85 Linköping, Sweden; ^4^Department of Pathology, Linköping University Hospital, 581 85 Linköping, Sweden

## Abstract

Littoral cell angioma is a rare vascular tumor of the spleen. The pathogenesis is unknown but the lesion is associated with several malignancies and immunological disorders. The diagnosis requires histopathological examination. The malignant potential of this lesion is unknown, which is why splenectomy is recommend for all cases. Symptomatic cases generally suffer from hypersplenism and pyrexia. A previously healthy 20-year-old female was diagnosed with colonic Crohn's disease; as part of the work-up a magnetic resonance enterography was performed which showed multiple signal changes of the spleen. The patient reported chronic abdominal pain in the left upper quadrant, malaise, and fever. The unknown splenic lesions prompted a laparoscopic splenectomy; pathology revealed a littoral cell angioma. The abdominal pain and malaise remitted but the fever persisted one year despite adequate treatment of the patient's Crohn's disease. Littoral cell angioma is associated with immune-dysregulation including Crohn's disease with several reported cases. Signs and symptoms of hypersplenism and splenic lesions on imaging should raise suspicion of littoral cell angioma in patients with Crohn's disease. Magnetic resonance enterography to assess disease severity in Crohn's disease may provide an opportunity to study the prevalence and natural history of this rare splenic tumor.

## 1. Introduction

Littoral cell angioma, LCA, is a primary vascular tumor of the spleen, first described by Falk et al. in 1991 [1]. It is a rare neoplasm which is thought to arise from the splenic cords showing both endothelial and histiocytic phenotype [1]. It has been associated with both malignancies [2] and immune-dysregulation [3]. Around 150 cases have been reported and although radiological features have been described for US, MRI, and CT, findings are unspecific and diagnosis usually requires histopathology [4] on image guided biopsy specimen [5] or splenectomy specimen. The LCA is frequently an incidental finding on imaging but some cases are symptomatic. Symptomatic cases typically develop signs and symptoms of hypersplenism including anemia, splenomegaly, and thrombocytopenia as well as constitutional symptoms including fever [3]. Treatment is splenectomy as it cures symptoms and malignant lesions cannot be excluded with imaging [6]. We report a case of LCA in a patient with new onset of Crohn's disease, naive to biologics, and with little exposure to immunosuppressive agents before the incidental finding of LCA on magnetic resonance enterography, MRE.

## 2. Case Presentation

A 20-year-old, previously healthy female with a two-month history of abdominal pain, bloody diarrhoea, and tenesmus was referred to the Gastroenterology Clinic at the Linköping University Hospital by her primary care physician. She underwent colonoscopy that revealed a friable erythematous area with contact bleeding that spanned from the splenic flexure and 15 cm down the descending colon; biopsies were taken throughout the procedure. These findings were interpreted as Crohn's disease because of the distribution of inflammation although histology-pathology showed unspecific inflammation more closely related to ulcerative colitis. The patient was started on prednisolone which was tapered as planned over 8 weeks. Follow-up with fecal calprotectin showed no response and the patient was started on balsalazide.

**Figure 1:**
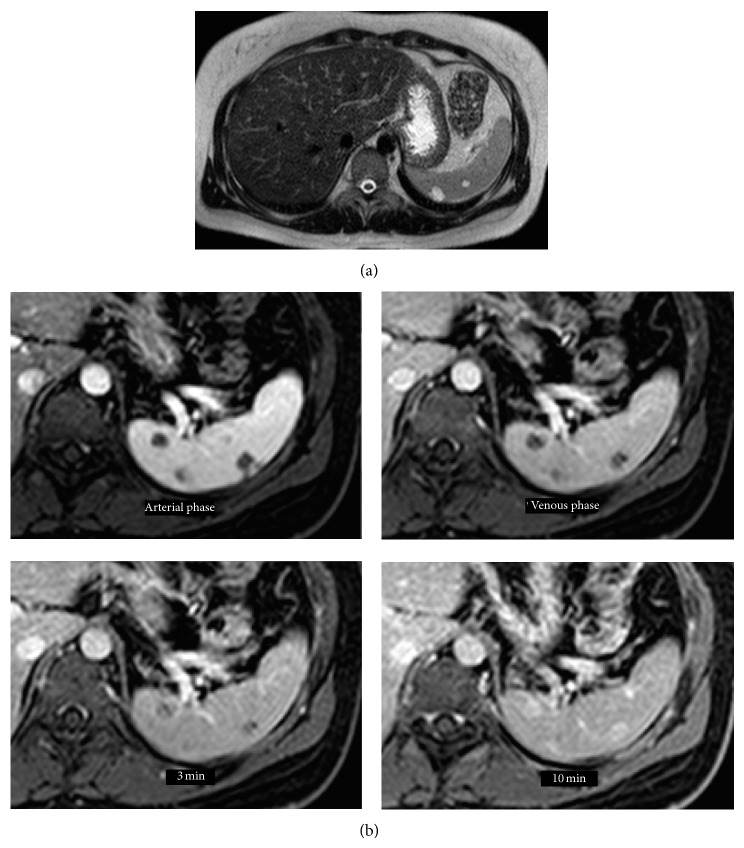
(a) Multiple lesions throughout the spleen up to 15 mm in size. They have a high signal on T2 and SPIR. (b) Signal intensity was low on T1 sequences. After gadolinium contrast, no attenuation was seen in the arterial phase, heterogeneous contrast enhancement in the portal venous phase, and after 3 minutes the enhancement is stronger in the periphery than central in the lesions. After 10 minutes the signal is stronger in the lesions than the surrounding splenic tissue. The signal pattern is in keeping with hemangiomas. On histopathological examination the diagnosis of LCA was confirmed.

When the patient came for follow-up at 9 months she suffered from fever and bloody diarrhoea once daily. This was regarded as colonic Crohn's disease and budesonide was added to balsalazide, An MRE was made one month after the follow-up and it revealed no small bowel engagement; however it showed multiple signal changes in the spleen, benign cysts where suspected. A contrast enhanced ultrasound was made that showed indentations in the superior portion of the spleen as well as multiple minor cystic lesions and a central 1 cm lesion with late contrast loading, possibly a hemangioma. After two months the effect of budesonide wore off and the patient again suffered from bloody diarrhoea. Sigmoidoscopy showed proctitis with diminished vascularity and contact bleeding. Budesonide was switched to mesalamine which had no effect; therefore azathioprine was tried but a colonoscopy one month later showed inflammation from the sigmoid colon till the splenic flexure. The MRE images were once again examined and it was decided that an MRI was necessary to establish the nature of the splenic lesions. The MRI showed multiple lesions with a contrast pattern of hemangiomas (Figures 1(a) and 1(b)). Two months after the MRI she presented acutely to the gastroenterology outpatient clinic with sudden worsening of abdominal pain that had been present for one month. There were no associated GI or micturition symptoms, body temperature of 38, 3°C with abdominal tenderness in the upper left quadrant, no guarding, rigidity, or mass.

**Figure 2:**
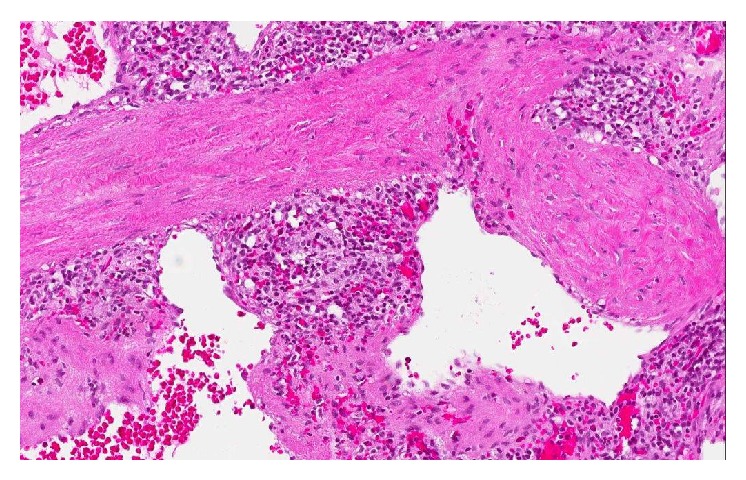
Photomicrograph ×20 magnification. Littoral cell angioma of the spleen composed of small anastomosing and irregular channels reminiscent of splenic sinuses and covered of neoplastic plump “littoral” cells with low mitotic activity.

**Figure 3:**
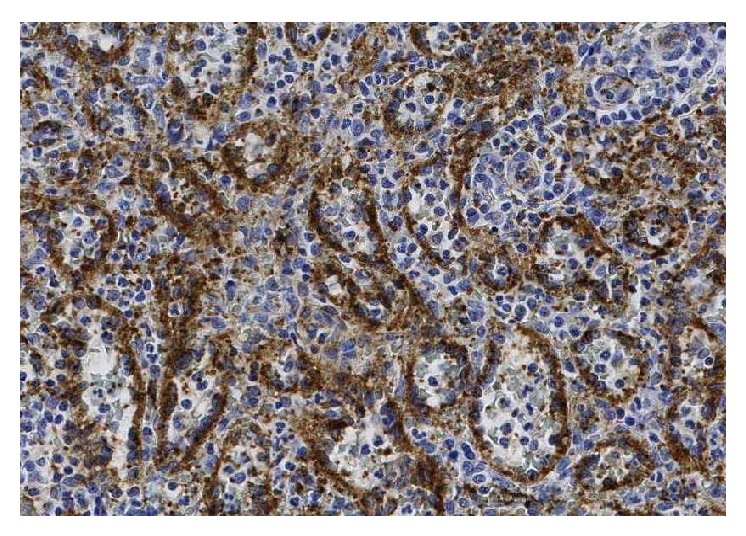
Photomicrograph ×20 magnification. The neoplastic cells express FVIII.

**Figure 4:**
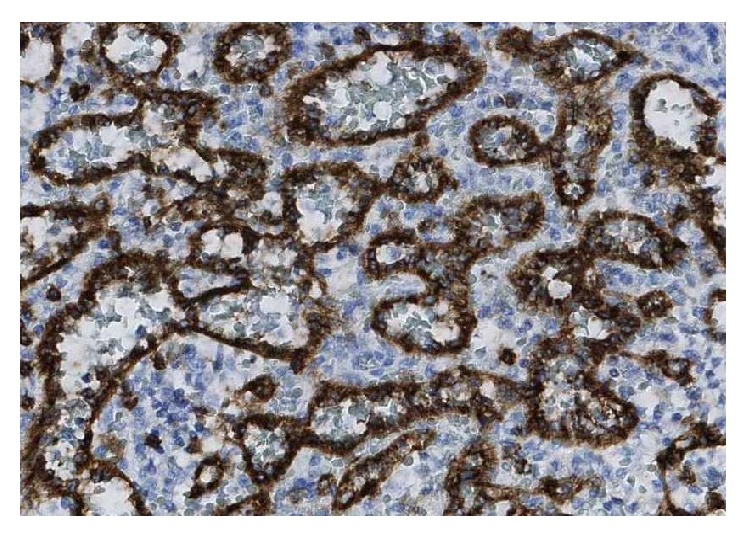
Photomicrograph ×20 magnification. The neoplastic cells express histiocytic antibody CD 68 as well as VIII whereas usual endothelial splenic cells only express VIII.

The patient was discussed at the upper gastrointestinal interdisciplinary round 2 months later. LCA or similar benign tumor was suspected and splenectomy was favoured due to the patient's symptoms. A laparoscopic splenectomy was performed, after adequate immunisation, without complications 3 months later. The patient got better and could eventually resume working. Repeated fecal calprotectin was negative. The pathology report confirmed LCA of the spleen (Figures 2, 3, and 4). The episodic fever persisted for one year and has been extensively investigated. A rheumatology consult (the patient reported joint pain and stiffness of the hand and has a first-degree relative with rheumatoid arthritis), capsule endoscopy and laboratory tests for VIP, pancreatic polypeptide, and gastrin all came back negative. After one year the fever gradually disappeared.

## 3. Discussion

Several cases link Crohn's disease to LCA [1, 7, 8]. LCA should be suspected in patients with Crohn's disease who develop signs and symptoms of hypersplenism or where imaging shows lesions of the spleen. Azathioprine, corticosteroids, and other immunosuppressive drugs have also been implicated [3]. In our patient the LCA was found on MRE before azathioprine treatment was initiated. The courses of prednisolone, budesonide, and balsalazide prior to MRE were all relatively short. In the cases where LCA has been tied to immunosuppression, patients have been treated for years [9–11].

TNF-*α* has been suggested to play a role in the pathogenesis of LCA, both in itself in the presence of immune-dysregulation [12] and in immunosuppression with anti-TNF-*α* agents [10]. Since our patient has not been exposed to biologics it further strengthens the hypothesis that LCAs are associated with immune-dysregulation. It is also to our knowledge the first case where systemic symptoms persisted despite splenectomy. The episodic pyrexia points towards an immunologic component of the disease. Why only some patients develop systemic symptoms remains elusive. The pyrexia has been attributed to the patients LCA since a comprehensive work-up failed to find a cause and her Crohn's disease was in remission, repeated fecal calprotectin negative.

The LCA in our patient was discovered on MRE before the onset of symptoms. Imaging is an important part of the assessment of disease severity in Crohn's disease; this generates a lot of data. These images may provide an opportunity to study the natural history and prevalence of LCA. Since Crohn's disease seems to be a risk factor for LCA, patient cohorts may be sought through reexamination of imaging studies from patients with Crohn's disease.

## 4. Conclusion

LCA is a rare neoplasm associated with immune-dysregulation. Several cases link LCA with Crohn's disease. Our case is the first where pyrexia, not explained otherwise, persisted for a year after splenectomy further suggesting an immunologic association. Splenic nodules on imaging and hypersplenism should always raise suspicion of LCA in patients with Crohn's disease. The MRE studies of patients with Crohn's disease may provide an opportunity to study the natural history and prevalence of LCAs in patients with Crohn's disease.

## References

[1] Falk S., Stutte H. J., Frizzera G. (1991). Littoral cell angioma: a novel splenic vascular lesion demonstrating histiocytic differentiation. *The American Journal of Surgical Pathology*.

[2] Bisceglia M., Sickel J. Z., Giangaspero F., Gomes V., Amini M., Michal M. (1998). Littoral cell angioma of the spleen: an additional report of four cases with emphasis on the association with visceral organ cancers. *Tumori*.

[3] Harmon R. L., Cerruto C. A., Scheckner A. (2006). Littoral cell angioma: a case report and review. *Current Surgery*.

[4] Grantham M., Einstein D., McCarron K., Lichtin A., Vogt D. (1998). Littoral cell angioma of the spleen. *Abdominal Imaging*.

[5] Shah S., Wasnik A., Pandya A., Bude R. O. (2011). Multimodality imaging findings in image-guided biopsy proven splenic littoral cell angioma: series of three cases. *Abdominal Imaging*.

[6] Abbott R. M., Levy A. D., Aguilera N. S., Gorospe L., Thompson W. M. (2004). Primary vascular neoplasms of the spleen: radiologic-pathologic correlation. *Radiographics*.

[7] Blansfield J. A., Goldhahn R. T., Josloff R. K. (2005). Littoral cell angioma of the spleen treated by laparoscopic splenectomy. *Journal of the Society of Laparoendoscopic Surgeons*.

[8] Sarandria J. J., Escano M., Kamangar F., Farooqui S. O., Montgomery E., Cunningham S. C. (2014). Littoral cell angioma: gastrointestinal associations. *Gastrointestinal Cancer Research*.

[9] Tan Y. M., Chuah K. L., Wong W. K. (2004). Littoral cell angioma of the spleen. *Annals of the Academy of Medicine Singapore*.

[10] Cappello M., Bravatà I., Cocorullo G., Cacciatore M., Florena A. M. (2011). Splenic littoral cell hemangioendothelioma in a patient with crohn's disease previously treated with immunomodulators and anti-TNF agents: a rare tumor linked to deep immunosuppression. *American Journal of Gastroenterology*.

[11] Tholouli E., Roulson J.-A., Byers R., Burton I., Liu Yin J. A. (2003). Littoral cell angioma of the spleen in a patient with severe aplastic anaemia. *Haematologica*.

[12] Cordesmeyer S., Pützler M., Titze U., Paulus H., Hoffmann M. W. (2011). Littoral cell angioma of the spleen in a patient with previous pulmonary sarcoidosis: a TNF-*α* related pathogenesis?. *World Journal of Surgical Oncology*.

